# Histological differences in the adherence of connective tissue to laser-treated abutments and standard abutments for dental implants. An experimental pilot study in humans

**DOI:** 10.4317/medoral.21949

**Published:** 2017-10-21

**Authors:** Mónica Blázquez-Hinarejos, Raúl Ayuso-Montero, José-Manuel Álvarez-López, Maria-Cristina Manzanares-Céspedes, José López-López

**Affiliations:** 1 Master of Medicine, Surgery and Oral Implantology.University of Barcelona, Faculty of Dentistry, Barcelona, Spain; 2 Prosthodontics Unit. University of Barcelona, Faculty of Dentistry, Barcelona, Spain; 3 Oral Health and Masticatory System Group (Bellvitge Biomedical Research Institute) IDIBELL, L’Hospitalet de Llobregat, Barcelona, Spain; 4 Commonwealth Scientific and Industrial Research Organisation (CSIRO). Canberra, Australia; 5 Human Anatomy and Embryology Unit. University of Barcelona, Faculty of Dentistry, Barcelona, Spain; 6 Growth factors and cell differenciation (Bellvitge Biomedical Research Institute) IDIBELL, L’Hospitalet de Llobregat, Barcelona, Spain; 7 Oral Medicine Unit. University of Barcelona, Faculty of Dentistry, Barcelona, Spain. // Chief Medical Surgical Service. Dental Hospital.University of Barcelona

## Abstract

**Background:**

The goal of the current study is to assess the difference in connective tissue adherence to laser microtextured versus machined titanium abutments.

**Material and Methods:**

Six patients were selected and each of them received 2 implants, one combined with a laser treated abutment and one with a machined abutment. After three months, the abutments were retrieved together with their surrounding gingival tissue for histological analysis. Qualitative and quantitative evaluation of microscopical images was performed to assess the presence or absence of adherence between the soft tissues and the abutment, and the percentage of soft tissue adhered to the two different surfaces.

**Results:**

Intimate adherence between connective tissue and the laser treated abutments, while on machined abutments no adherence was detected. A significant difference was found in the percentage of surface in contact with soft tissue between both implant abutments *p*=0.03.

**Conclusions:**

Within the limitation of the current study, it can be concluded that connective tissues show enhanced adherence to microtextured abutments compared to machined abutments.

** Key words:**Abutment, connective tissue, dental implant, gingiva, human, laser.

## Introduction

Oral rehabilitation on osseointegrated dental implants is a highly predictable technique for the restoration of partially or totally edentulous patients (1-3). Achievement of implant stability and maintenance of crestal bone levels are prerequisites for a successful long-term function of dental implants. Some researchers regard infection (i.e. peri-implantitis) as the cause of virtually all bone loss, whereas others see crestal bone loss as an unavoidable phenomenon following surgery and implant loading (4). Two of the factors associated with peri-implant bone loss are implant overload and bacterial proliferation (5). Collars have been modified to include a microtextured section that will enhance soft tissue attachment to the implant’s cervical area. This soft tissue seal is believed to help with peri-implant infection and bone loss (6). A number of pre-clinical and human studies, have shown the attachment of connective tissues to laser microtextured implant collars and the formation of a biologic seal (7-11). In fact, it has been proven that there is less bone loss around microtextured implant collars over time than in smooth implant collars (12).

Those findings sparked an interest to move the biologic seal and connective tissue attachment up to the abutment section. Nevins *et al.* performed a study on an animal model using laser microtextured and machined abutments on non lased implants and found a better connective tissue attachment around lased abutments compared to machined ones. Functionally oriented rather than parallel connective tissue fibers apposed lased abutments (13).

The goal of the current study is to assess the difference in connective tissue adherence to laser microtextured versus machined titanium abutments in a split mouth study with human histology.

## Material and Methods

All procedures and materials used in the present study were authorized by the Ethics Committee for Clinical Research of the University of Barcelona (CEIC # 09/2012). All participants were informed about their participation in the study and signed an informed consent. The study followed the guidelines of the Declaration of Helsinki on Medical Research involving Human Subjects and was registered in ClinicalTrials.gov with the following ID: NCT01954485.

- Patient selection:

Patients in need of two implants each were enrolled in the study, all of them treated at the Master of Medicine, Surgery and Oral Implantology at the School of Dentistry of the University of Barcelona. The patients included showed a good systemic health status (ASA I-ASA II) (14) and had at least 7 mm of keratinized gingiva in the bucco-lingual direction. The excluding factors were: smoking habit, physical, systemic or psychological conditions which contraindicated a surgical intervention and need of additional surgical techniques for implant placement, such as bone grafting or soft tissue regeneration procedures.

All implants were inserted in non-aesthetic posterior sections, unilateral or bilateral but not contiguous, because the soft tissue would have created a vast injury section when retired.

- Surgical procedure:

All patients received antibiotic coverage with 2g of amoxicillin 1 hour prior to the intervention and 2g per day during the following 7 days (15). In each patient, two implants were surgically placed under local anesthesia (Ultracain®, epinephrine 1:50.000, Normon, Madrid, Spain). Crestal incisions were performed and full thickness flaps were raised with #12 surgical blades (Braun®, Melsungen, Germany). The implants inserted were BioHorizons Internal Implants® (BioHorizons, Birmingham, USA). Each patient received one 3inOne® machined titanium abutment (BioHorizons, Birmingham, USA) that has 8 mm height, and one simple solution abutment with Laser-Lok® (BioHorizons, Birmingham, USA), that has 6 mm height and 0.7 mm of laser-treated section in the closest area to the prosthetic connection.

**Figure 1:**
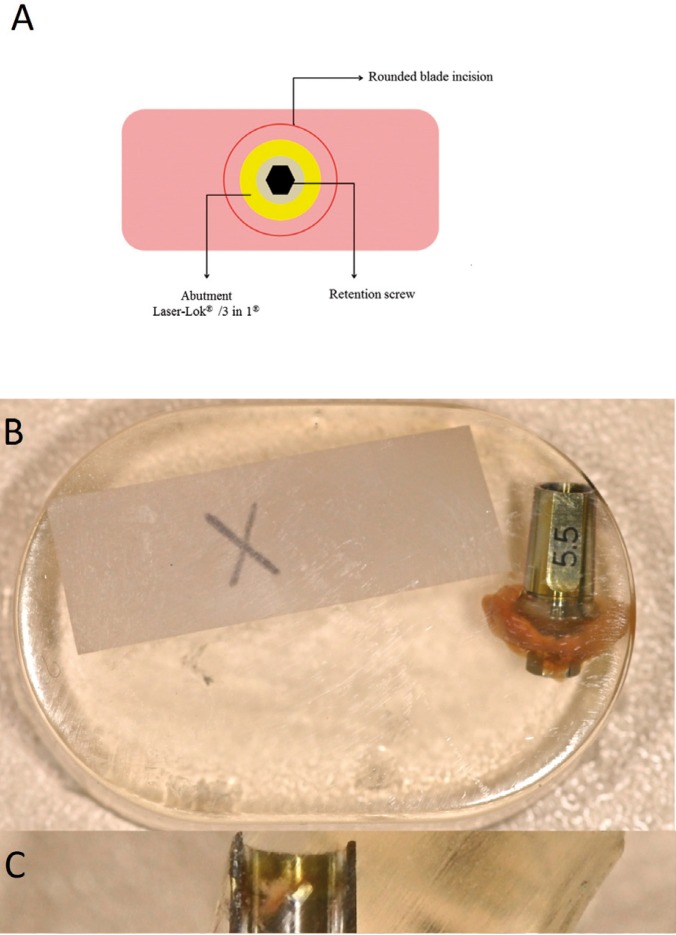
(A) Sample obtention scheme; (B) Sample embedded in light-cured resin; (C) Light-cured resin block cut in half.

A minimum distance 1.5 mm between implants and adjacent teeth were maintained to preserve surrounding soft tissues and bone (16). Suturing was performed with non-resorbable suture material (4-0 silk, Aragó®, Barcelona, Spain). As adjunctive treatment, chlorhexidine 0.12% mouth rinses were prescribed for 14 days. After 14 days, a post-op recall appointment was held for suture removal and wound check. Patients were subjected to clinical recall appointments after 30 and 60 days. Ninety days after the first intervention, a second surgery was carried out. The incision was made with a concentrically positioned punch 2 mm larger than the diameter of the abutment to establish direct contact between the punch blade and the bone surface (Fig. 1A). After the incision, the abutment was unscrewed, removing a complex formed by the abutment and the surrounding 1 mm of gingival tissue for histological assessment. After removing the abutment, a Laser-Lok® abutment was placed on all of the implants and the soft tissues were allowed to heal for 15 weeks following the usual prosthetic protocol (17).

- Randomization:

The present study is a single-blind randomized controlled pre-clinical trial. The abutments were assigned to each of the implants through the SPSS 15.0 software program (SPSS Inc, Chicago, Il, USA). The patients, the laboratory technician and the person in charge of examining the samples did not know the type of abutment associated with each sample. The person who places the implants and abutments assigns to each sample a randomized numeric code provided by the SPSS 15.0 software and registers the type of abutment which corresponds to each code.

- Histologic preparation:

Samples were immersed in formaldehyde 10% solution and then processed for evaluation using the methacrylate embedding technique described by Donath (18). First, the samples were dehydrated with different concentrations of alcohol under constant agitation. Plastic infiltration was performed mixing glycol methacrylate (Technovit 7200®, VLC - Heraus Kulzer GMBH, Werheim, Germany) and 1% benzoyl peroxide (BPO®: Heraus Kulzer GMBH, Werheim, Germany) with ethyl alcohol at different concentrations, finishing with two infiltrations of pure glycol methacrylate. The samples were embedded under vacuum conditions in light-cured resin (Technovit 7200®) (Fig. 1B). The resulting blocks were cut in half with a band-saw (Exakt 300) following the long axis of the abutment, under irrigation (Fig. 1C). Each section obtained was mounted on an acrylic slide with the help of a resin (Technovit 4000®Heraus Kulzer GMBH, Werheim, Germany), using a vacuum adhesive press. All acrylic slides were subjected to microgrinding and polishing (Exakt Micro Grinding System®, Apparatebau GMBH, Hamburg, Germany) with silicon carbide papers of different thicknesses until achieving slides of 38 µm. Two of the sections were selected to be stained, one with Masson-Goldner trichrome stain and the other with toluidine blue. The samples were assessed under a Leica DMD optical microscope (Leica Microsystems, Wetzlar, Germany), and digital microscopic images were obtained to assess the presence or absence of adherence between the soft tissues and the abutment, centering the objective on the first 0.7mm of the abutments collar (treated zone in the Laser-Lok abutments and non-treated on the mechanised abutments), and focusing different magnification on the same zone.

**Figure 2:**
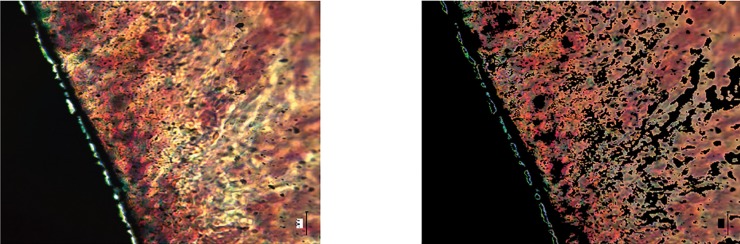
Image binarization and automatic selection of the color threshold between the abutment and tissue for calculating the TAC.

Modifying a previous method described to calculate the bone-implant-contact (19), an algorithm was developed using MATLAB to automatically binarize the images and define the color threshold for the titanium and the tissue, calculating the tis-sue-abutment-contact (TAC) as the percentage of abutment surface with tissue contact (Fig. 2) in the portion adjacent to the prosthetic connection.

- Statistical analysis:

The presence of adherence between the connective tissue and the abutment surface was treated as a dichotomous qualitative variable in the qualitative analysis. Accepting an alpha risk of 0.05 and a beta risk of 0.2 in a two-sided test, 18 subjects were calculated to be necessary to recognize as statistically significant a difference consisting in an initial proportion of 0 and a final proportion of 0.5. A contingency table was created and Clopper-Pearson test was applied to compare the connective tissue adherence proportion with each of the procedures. The null hypothesis was that the abutment proportion with intimate adherence of the connective tissue was the same on the Laser-Lok-treated ones as on the machined ones. The quantitative study on the TAC percentage was analyzed using the Wilcoxon test. In both statistic test a 0.05 level of significance was considered. The SPSS for Windows v23.0 software (IBM Statistics, Chicago, Il, USA) was used for these purposes.

## Results

Nine patients took part in the study, both samples from one patient were lost, and two more samples were lost from two different patients. All of them during their preparation. The complimentary samples obtained from these two patients were discarded in the study. The results summarize the participation of six patients, four males and two females, between 40 and 76 years of age (mean 58 years), recruited during the first 4 months of the study. In all patients, healing was successful and uneventful, and all of the cases were restored with a definitive prosthesis.

**Figure 3:**
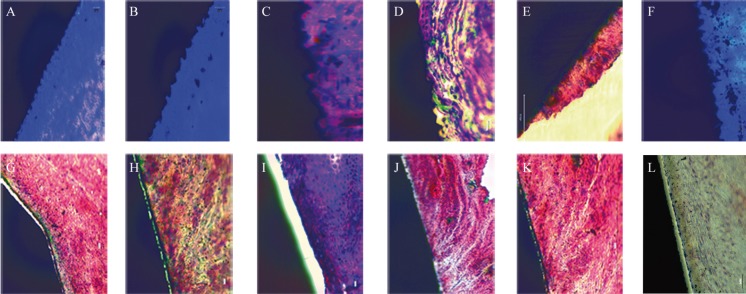
Histological images of the six samples obtained A through F Laser-Lok® abutments, G through L3inOne® abutments. Scale bars represent 20µm. Stain with Masson-Goldner trichrome stain and toluidine blue.

- Histologic observation:

The qualitative analysis of the images showed that in the six samples of Laser-Lok® abutments, an adherence of the soft tissues to the prosthetic abutment was observed (Figs. 3A through 3F). In none of the samples of 3inOne® machined abutments soft tissue adherence was observed (Figs. 3G through 3L).

On the Laser-Lok® abutments the soft tissue was closely adhered to the treated zone then the tissue abruptly separated when the treated zone ended and the machined zone started, therefore the soft tissue had two different behaviors on the same abutment. The results of the Clopper-Pearson test are presented in  Table 1. The observed differences are statistically significant with a CI of 95%.

**Table 1:**
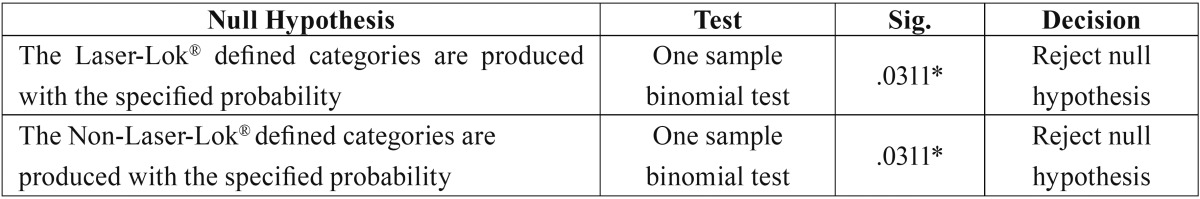
Clopper-Pearson test results..

The mean percentage of TAC on Laser-Lok® abutment was 98.8%, and 24.1% on the 3inOne. The TAC quantitative analysis results are shown in  Table 2. The Wilcoxon test showed significant differences with *p*=0.03.

**Table 2:**
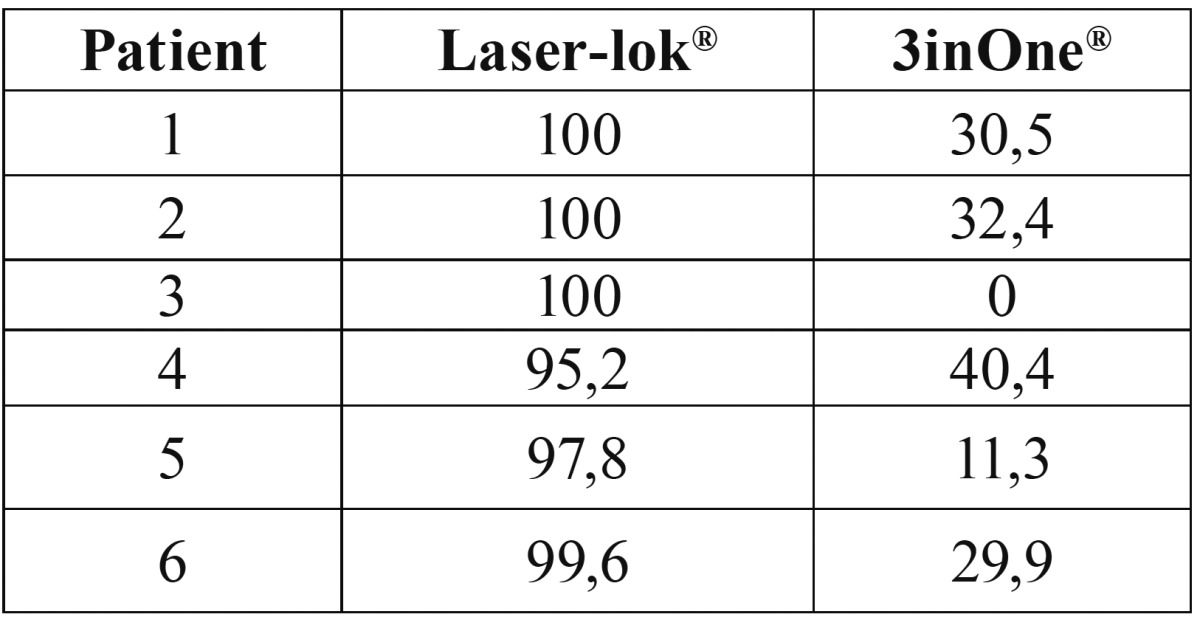
Tissue abutment contact (TAC) in % of the two abutment surfaces.

## Discussion

The present study was designed to assess the presence or absence of connective tissue adherence on definitive prosthetic abutments treated with the Laser-Lok® technology. Volunteers were recruited during 4 months and followed up for at least 12 months after placement of the definitive prosthetic restoration. In all the samples obtained from the 3inOne® machined abutment group (control) no adherence was found between the surrounding soft tissues and the prosthetic abutment, and in all samples obtained from simple solution abutments with Laser-Lok® soft tissue adherence to the abutment was found. The statistical significance of the results in these 6 participants, did researchers stop the sample recruiting and present these results as a pilot study.

An in vitro study comparing cell morphology and proliferation on Laser-Lok®, titanium, and zirconia surfaces showed that the morphology of cells (observed under an electron microscope) attached to Laser-Lok® surfaces was significantly different from that on other surfaces. The majority of cells in the Laser-Lok® group were elongated and had pseudopods (20). Assessment of cell morphology is important to determine the cell-surface affinity. Cells with an elongated morphology can more strongly attach to surfaces as a result of their cytoplasmic pseudopods compared to round cells (20). This could explain the reason why the machined abutments in our study had no surrounding soft tissue adherence even though the biopsy method was the same for all samples.

The presence of a wide band of keratinized mucosa (at least 7mm) was necessary to ensure proper reintegration of the gingival tissue after sample collection. This will allow a long-term stability of the keratinized gingival (21-22). Nevins *et al.* revealed the reintegration of connective tissue after 15 weeks around a laser treated abutment (17,23). Other recent pre-clinical studies showed that when using a standard smooth surface healing abutment an overall superior soft tissue attachment to the abutment surface is achieved by de-epithelializing the crevice and replacing the abutment with a Laser-Lok® one (24). For this reason, all implants were definitively restored with Laser-Lok® abutments.

Sections of all abutments were obtained for staining with toluidine blue, as in the studies by Nevins (7,13,17,23), and with Masson-Goldner trichrome stain, as in the study by Schwarz (25,26). As different cuts were obtained from the same sample, both staining procedures were used to assure the detection of connective tissues.

Finally, within the limitation of the current study, it can be concluded that connective tissues show enhanced adherence to microtextured abutments compared to machined abutments.
